# Molecular imaging of the urokinase plasminogen activator receptor: opportunities beyond cancer

**DOI:** 10.1186/s13550-020-00673-7

**Published:** 2020-07-28

**Authors:** V. M. Baart, R. D. Houvast, L. F. de Geus-Oei, P. H. A. Quax, P. J. K. Kuppen, A. L. Vahrmeijer, C. F. M. Sier

**Affiliations:** 1 Department of Surgery, Leiden University Medical Center, Albinusdreef 2, 2333 ZA Leiden, The Netherlands; 2 Department of Radiology, Section of Nuclear Medicine, Leiden University Medical Center, Leiden, The Netherlands; 3 Biomedical Photonic Imaging Group, University of Twente, Enschede, The Netherlands; 4 Percuros BV, Leiden, The Netherlands

**Keywords:** Nuclear imaging, PET, Image-guided surgery, uPA

## Abstract

The urokinase plasminogen activator receptor (uPAR) plays a multifaceted role in almost any process where migration of cells and tissue-remodeling is involved such as inflammation, but also in diseases as arthritis and cancer. Normally, uPAR is absent in healthy tissues. By its carefully orchestrated interaction with the protease urokinase plasminogen activator and its inhibitor (plasminogen activator inhibitor-1), uPAR localizes a cascade of proteolytic activities, enabling (patho)physiologic cell migration. Moreover, via the interaction with a broad range of cell membrane proteins, like vitronectin and various integrins, uPAR plays a significant, but not yet completely understood, role in differentiation and proliferation of cells, affecting also disease progression. The implications of these processes, either for diagnostics or therapeutics, have received much attention in oncology, but only limited beyond. Nonetheless, the role of uPAR in different diseases provides ample opportunity to exploit new applications for targeting. Especially in the fields of oncology, cardiology, rheumatology, neurology, and infectious diseases, uPAR-targeted molecular imaging could offer insights for new directions in diagnosis, surveillance, or treatment options.

## Background

Tissue remodeling is pivotal in embryonic development, tissue repair, and numerous pathologies. Temporary degradation of the extracellular matrix (ECM) is a delicate process requiring the careful coordination of proteases, receptors, and cell-signaling molecules where over-degradation can result in osteoarthritis, osteolysis, cardiomyopathy, and invasion/metastasis of tumor cells, and where over-production of the ECM often leads to fibrosis [1]. It seems conceivable that monitoring of the process of matrix remodeling offers possibilities for diagnosis, surveillance, and possibly even treatment of the associated diseases. For clinical applications, such as biomedical imaging or therapy, a cell-associated target protein with a central role within the ECM-remodeling process, but with limited expression in healthy tissue, would be helpful in identifying patient groups requiring more intensive monitoring or therapy. Furthermore, molecular imaging enables real-time imaging of pathophysiology, providing novel insights into disease processes that cannot be gathered with current techniques such as post-mortem tissue analysis or with animal models [2, 3].

Inherent to its nature, molecular imaging is fundamentally dependent on identifying appropriate targets that are informative about the underlying pathophysiology of the process studied [4]. As targeting different epitopes on the same protein may influence the ability to image specific processes, formal description of the epitope is crucial. Important to realize is that differing epitopes on the same protein can alter the results and consequently, describing the epitope of interest is just as crucial [5, 6]. Therefore, a key competence of targeted imaging is designing the best performing probe for the imaging modality of choice. The choices to be made are extensive and have already been covered in reviews elsewhere [7–9].

The urokinase plasminogen activator receptor (uPAR) holds a central position in ECM proteolysis, but, next to the proteolytic role, uPAR is also involved in cell-cell and cell-ECM interactions, regulating cell signaling and hereby controlling cell proliferation, differentiation, and migration [10]. uPAR is normally hardly found in healthy tissue, but it is present in virtually all human malignancies, associated with disease aggressiveness, allowing tumors to escape their original boundaries [11, 12]. As a result, the field of uPAR-based oncological imaging is progressing rapidly and, not surprisingly, various positron-emission tomography (PET)-based molecular imaging clinical trials are currently being conducted for diagnosing aggressive cancers and determining cancer aggressiveness (NCT02755675, NCT02945826, and NCT03307460) [13, 14].

The last two decades have revealed that uPAR is not only a central orchestrator in oncology but also in processes ranging from neurology to auto-immune diseases [15, 16]. Likewise, by unraveling the various (patho)physiological processes uPAR contributes to novel opportunities to diagnose, treat, or monitor diseases have been revealed. The current review aims to identify non-neoplastic diseases where uPAR is of pathophysiological relevance and elaborate on the molecular imaging opportunities this provides.

### The urokinase plasminogen activator receptor: a central player in an extensive interactome

**Fig. 1:**
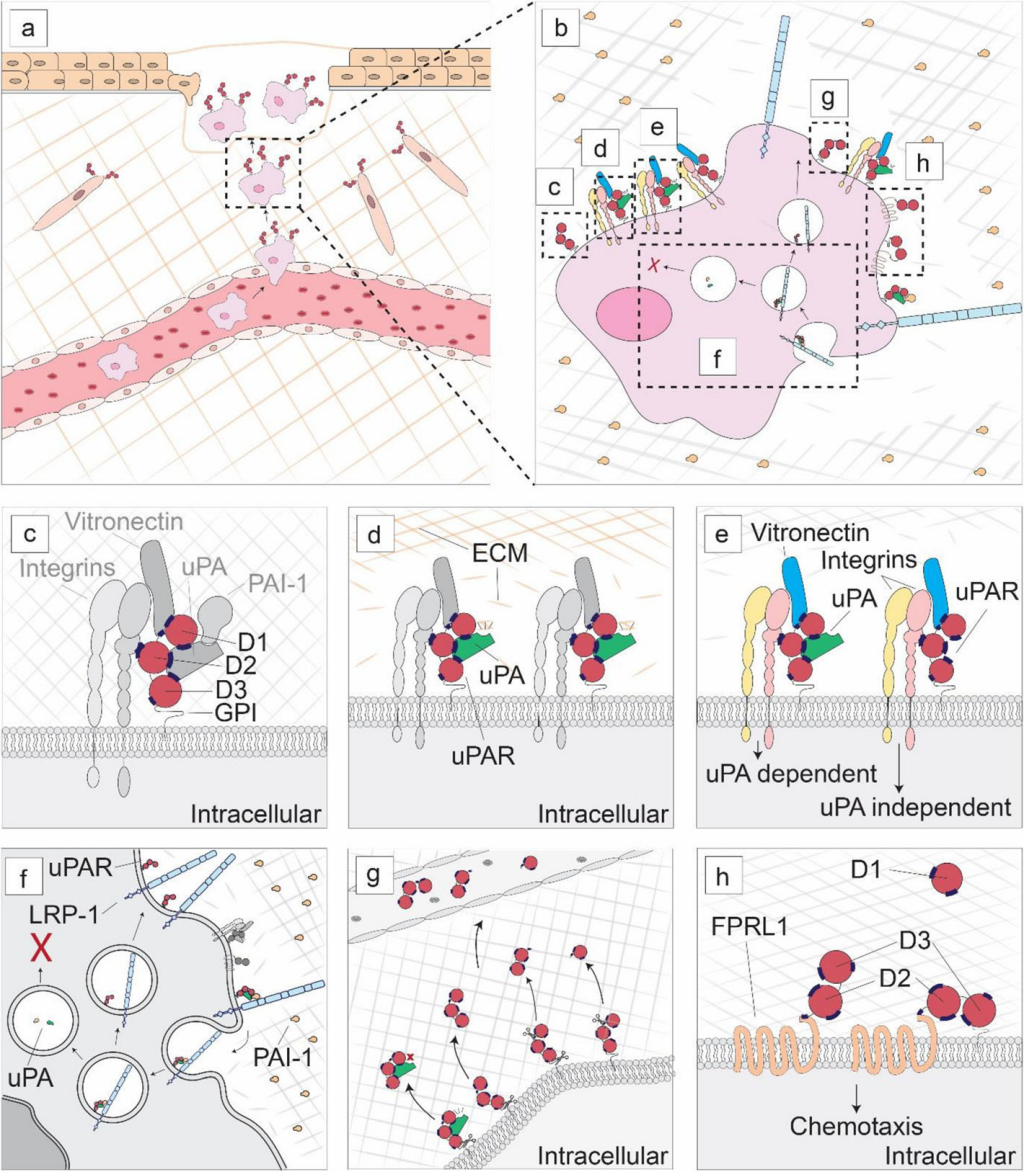
The urokinase plasminogen activator receptor (uPAR). **a** While usually quiescent in normal tissue, uPAR expression is observed transiently and locally during specific cellular processes such as extravasation and migration by wound healing. **b** At a cellular level, uPAR interacts in a multitude of pathways where the balance of each dictates the end result. **c** uPAR itself is a three domain extracellular structure linked to the plasma membrane by a glycosylphosphatidylinositol (GPI) anchor. **d** Classically uPAR functions as receptor for urokinase plasminogen activator (uPA) which subsequently breaks down the extracellular matrix (ECM) via plasminogen activation. **e** Intracellular signaling occurs via other receptors including vitronectin and integrins and can be uPA dependent and independent. **f** Internalization and recycling of uPAR occurs after a uPAR/uPA/PAI-1/LRP-1 complex has formed, which results in the degradation of uPA and PAI-1 and the recycling of uPAR and LRP-1. **g** uPAR can be cleaved at the GPI-anchor and between D1 and D2 resulting in various isoforms of soluble uPAR which can be quantified in the blood. **h** After cleavage of D1, uPAR D2-D3 induces chemotaxis by interacting with formyl peptide receptor-like 1 (FPRL1)

In 1985, uPAR was first identified on monocytes as the cell membrane receptor of the urokinase plasminogen activator (uPA) [17, 18]. In the following 35 years, uPAR has been identified, although often only expressed transiently, on, among others, fibroblasts, endothelial cells, epithelial cells, and neurons [11, 19]. Rather than being cell-specific, uPAR expression should be considered as process-specific with all cells being able to express uPAR, but only doing this at very specific events, such as the cell extravasation and migration observed during wound healing (Fig. 1a). Consequently, most cells at rest have no uPAR on their cell membrane [11]. A closer look at uPAR-expressing cells reveals that uPAR is implicated in multiple processes where the balance of this determines the end result (Fig. 1b).

To understand how uPAR can play such a diverse and central role, a careful examination of its structure needs to be made. uPAR is a 283 amino acid glycosylphosphatidylinositol (GPI)-anchored membrane protein consisting of three domains (D1-D3) linked by two flexible hinges (Fig. 1c) [20]. These three domains form a concave surface where uPA can bind [21]. Subsequently, uPAR mediates its other, non-proteolytic-related effects via protein interactions on the outer surface. D1 and the hinge region between D1 and D2 are vital for uPAR-vitronectin interactions, whereas various epitopes on D2-D3 interact with integrins, G protein-coupled receptors (GPCRs) and receptor tyrosine kinases like epidermal growth factor receptor, platelet-derived growth factor receptor, and insulin-like growth factor 1 receptor [22, 23]. With over 42 interacting proteins described, uPAR forms a central orchestrator of cell proliferation, differentiation, migration, and survival [10, 22].

Classically, the function of uPAR is fairly straightforward. Without intracellular or transmembrane domains, uPAR primarily functions as a receptor for (pro)uPA (Fig. 1d) [24]. uPA is a serine protease that catalyzes the activation of the ubiquitously present plasminogen into plasmin. Active plasmin degrades ECM proteins by itself or via activation of latent matrix metalloproteases (MMPs) [25]. Localization of both the inactive form, pro-uPA and active uPA to the cell surface, allows cells to focus extracellular matrix degradation toward the leading edge of the cell [26, 27]. However, this classic view of uPAR does not justify the many subtleties present in the uPAR interactome. For instance, the distinct central binding cavity of uPAR and the flexible hinges result in a conformational change after uPA binding that alters the vitronectin binding site, enhancing uPAR-vitronectin interaction on the outer surface of uPAR [23, 28–30]. Vice versa, vitronectin binding to uPAR influences the affinity for uPA [31]. Another subtlety of uPAR characteristics lies in the GPI anchoring to the cell, which influences distribution of uPAR toward lipid rafts and subsequently promotes specific protein-protein interactions [32–34]. Furthermore, GPI anchorage allows a rapid removal from the cell membrane, allowing a quick turnover and response time.

The intracellular signaling pathway initiated by uPAR, either enabled by uPA, with or without vitronectin, is still not entirely understood (Fig. 1e) [35–39]. On neutrophils and macrophages, CD11b/CD18 (MAC1, complement receptor 3 or αMβ2) colocalizes with uPAR and is essential for adhesion, migration, and phagocytosis [40–47]. In combination with the β1 integrin subunit, uPAR promotes differentiation, proliferation, adhesion, of epithelial and other cells and stimulates expression of uPA, uPAR, and MMPs, promoting extracellular proteolysis [38, 48–54]. Furthermore, β3-uPAR-mediated signaling enhances cell motility and invasion, while β6-uPAR interaction stimulates proliferation and cell differentiation [55–58].

Finally, recycling and cleavage of uPAR play an important role in cell functioning (Fig. 1f).

Plasminogen activator inhibitor-1 (PAI-1) and uPA are internalized for degradation via uPAR and lipoprotein receptor-related protein 1 interaction (LRP1) [59–61]. uPAR and LRP1 are recycled to the cell membrane ready for new interactions [62]. Cleavage of uPAR can occur at two sites: (I) within the GPI anchor by lipases, resulting in soluble uPAR (suPAR) and (II) between D1 and D2 resulting in cleaved uPAR (soluble D1 and soluble or membrane-bound D2-D3) (Fig. 1g) [63]. The exact function of full-length suPAR is unclear but suPAR might function as a scavenger protein for uPA, consequently competitively inhibiting cell surface proteolysis [64, 65]. Cleavage of D1 unveils an amino acid sequence (amino acids, 88-92) on D2-D3 that is unable to interact with integrins but interacts with GPCR formyl peptide receptor-like 1 (FPRL1), prompting migration (Fig. 1h) [66]. When cleaved, the same D2-D3 epitope induces chemotaxis in FPRL1-expressing cells [67, 68].

### uPAR in cardiovascular disease: determining plaque instability in atherosclerosis

Although significant improvements have been made in the management of cardiovascular disease, it is still a leading cause of death worldwide [69]. The current state-of-art diagnostic techniques, such as angiography or perfusion imaging, can accurately identify stenosis location and luminal occlusion in order to guide revascularization, however, fail to determine risk of rupture [70, 71]. Identifying these patients is the next challenging frontier in cardiovascular disease research: more than 50% of patients who die suddenly have no evident clinical symptoms and autopsy studies indicate that the majority of myocardial infarctions are caused by non-flow limiting lesions [72–74]. Based on its mechanistic role, molecular imaging of uPAR expression status might be an alternative and more targeted tool to improve the recognition of atherosclerotic plaques and the risk of rupture.

**Fig. 2:**
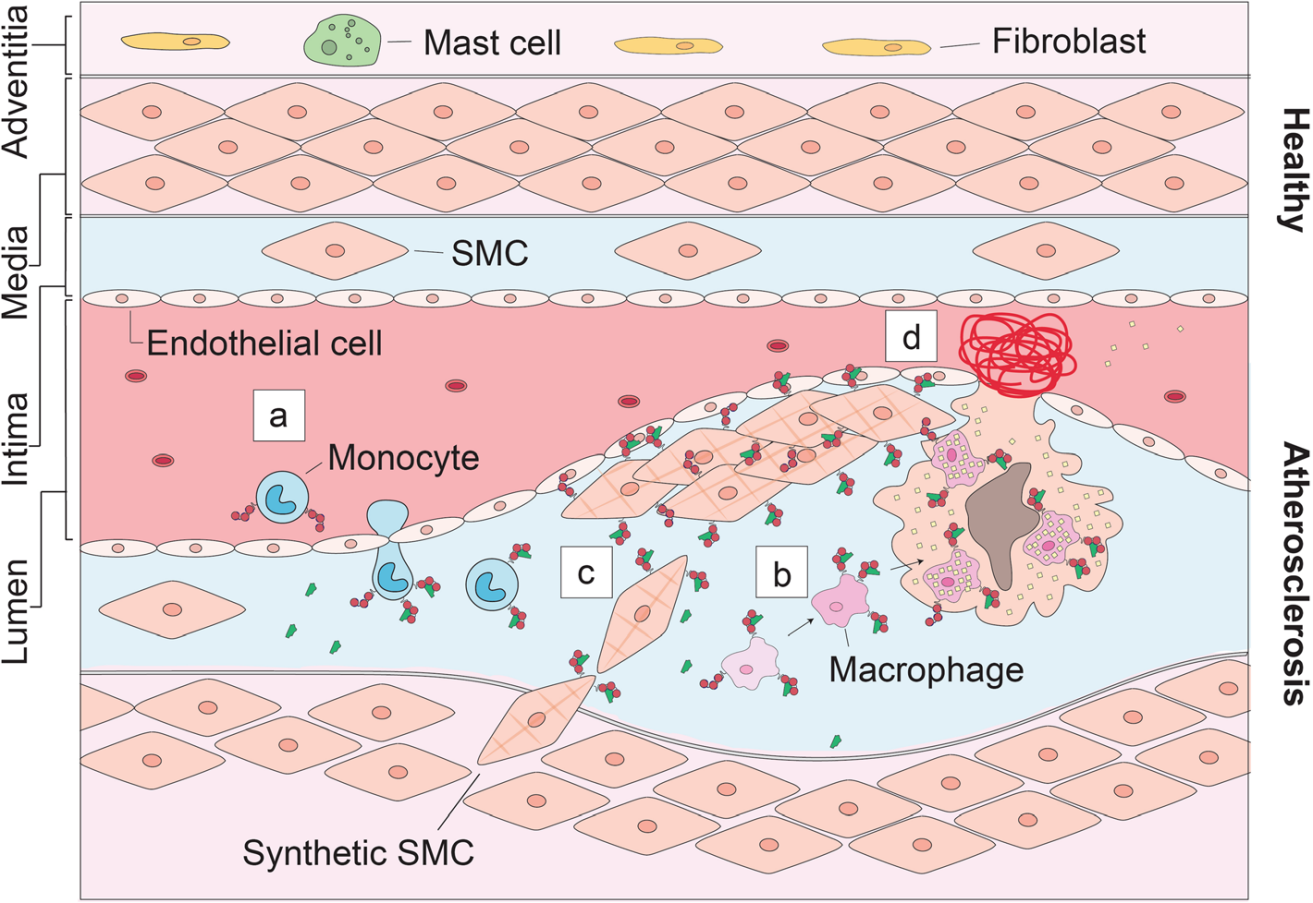
uPAR in atherosclerosis. **a** Monocyte extravasation across the endothelium lesions is dependent on uPAR. **b** Upon interaction with uPA these monocytes differentiate into macrophages, eventually resulting in cholesterol filled monocyte-derived macrophages. **c** uPA released from macrophages interacts with uPAR on synthetic smooth muscle cells stimulating their migration. **d** Localized uPAR overexpression in an atherosclerotic plaque increases the risk of rupture. uPAR is represented by the red 3-domain structure as described in Fig. 1 on the cell membrane of uPAR expressing cells and uPA by the green structure in the extracellular matrix and bound to uPAR

Atherosclerosis is the formation of intimal plaques consisting of two interacting regions: a central core covered by a fibrous cap. Cholesterol filled monocyte-derived macrophage-foam cells form the core whereas the cap consists of vascular smooth muscle cells (VSMCs) that have been recruited from the media [75–77]. In both regions of the plaques, the urokinase plasminogen activation axis (uPA/uPAR/PAI-1 axis) has been shown pivotal for development and progression of the disease. Monocyte adherence and recruitment toward lesion sites are dependent on uPAR expression, and upon arrival in the lesion, uPA interaction with uPAR has been implicated in the differentiation of monocytes to macrophages, and cholesterol biosynthesis and subsequent lipid uptake (Fig. 2a, b) [78–83]. In response to vascular injury, VSMCs undergo a change from a physiological contractile phenotype to the pathological synthetic phenotype, allowing them to migrate, proliferate, and produce extracellular matrix, as found in the caps of atherosclerotic plaques. This process is stimulated by intimal macrophages-derived uPA binding to the uPAR present on VSMCs (Fig. 2c) [84–93]. Furthermore, uPAR expression upregulates the calcification of these lesions, although the consequences for plaque stability remain to be clarified [94, 95]. Overall, many in vitro mechanistic studies demonstrate the enhanced presence and pivotal role of uPAR in atherogenesis and negative (inward) remodeling [78, 92, 96]. These data are supported by various immunohistochemical studies on patients, which have clearly localized uPAR overexpression to atherosclerosis: while normal arterial tissue is negative for uPAR, intensely positive stained lymphocytes, macrophages, and intimal smooth muscle cells are found in atherosclerotic lesions and atheroma’s [93, 97–101]. Likewise, the overexpression of uPAR is confirmed in gene analysis with a 1.5 fold higher uPAR expression in endarterectomies [99]. The level of uPAR overexpression has been associated with disease severity and localized uPAR expression is indicative for areas at risk for rupture (Fig. 2d) [98, 99, 102].

As uPAR has been implicated in the pathophysiology of atherosclerosis, various studies have attempted to improve disease outcomes by targeting of uPAR to block its function. Viral and non-viral expression vectors, encoding constructs consisting of ATF (the amino-terminal fragment of urokinase with high affinity for uPAR) in combination with inhibitors of the plasminogen pathway like BPTI (bovine pancreas trypsin inhibitor) or of matrix metalloproteinases like TIMP1 (tissue inhibitor of matrix metalloproteinases 1), successfully inhibited neointimal formation, VSMC migration, and vein graft thickening in rodent models and human saphenous vein cultures [103–106]. Eventually, a construct consisting of all three of these proteins has been shown to lead to the strongest reduction in vein graft thickening in hypercholesterolemic mice [107]. While these preclinical studies show evident potential of uPAR as target for atherosclerosis targeting, the concept has not yet been progressed toward a clinical application neither for therapy nor for diagnostic monitoring via molecular targeted imaging.

### uPAR in auto-immune disease: imaging disease activity in rheumatoid arthritis

Rheumatoid arthritis (RA) is a chronic inflammatory disease with a lifetime risk of 3.6% for women and 1.7% for men [108]. Anatomical imaging techniques, such as conventional radiology, ultrasound, and magnetic resonance imaging, along with clinical criteria, are the standard to diagnose and monitor RA [109]. These modalities are able to identify RA as soon as 6-8 weeks after arthritis onset and sometimes even before the first clinical symptoms [110, 111]. Current research efforts lie in patient stratification according to disease severity and identifying responders to expensive novel biologicals [111]. Targeted molecular imaging might offer a solution for the current goals of identifying aggressive disease and treatment potential, providing a more reliable prognosis, evaluating/comparing new therapies, and providing new insights in the pathophysiology of RA [110, 112].

As RA progresses, the initially sparsely populated articular region becomes infiltrated with immune cells, neutrophils and monocytes/macrophages, fibroblast-like synoviocytes (FLS), and osteoclasts [113, 114]. The interaction of these cells directly with each other and via cytokines has many similarities with locally invasive malignancies, leading to chronic inflammation, and tissue invasion, remodeling, and destruction [113, 115]. In the RA microenvironment, FLS acquire the tumor-like characteristic of being able to escape growth limits, enhance migration and invasion, and to prompt angiogenesis [16]. The similarities between RA and cancer have led to the identification of commonly activated pathways with one being centered around uPAR.

**Fig. 3:**
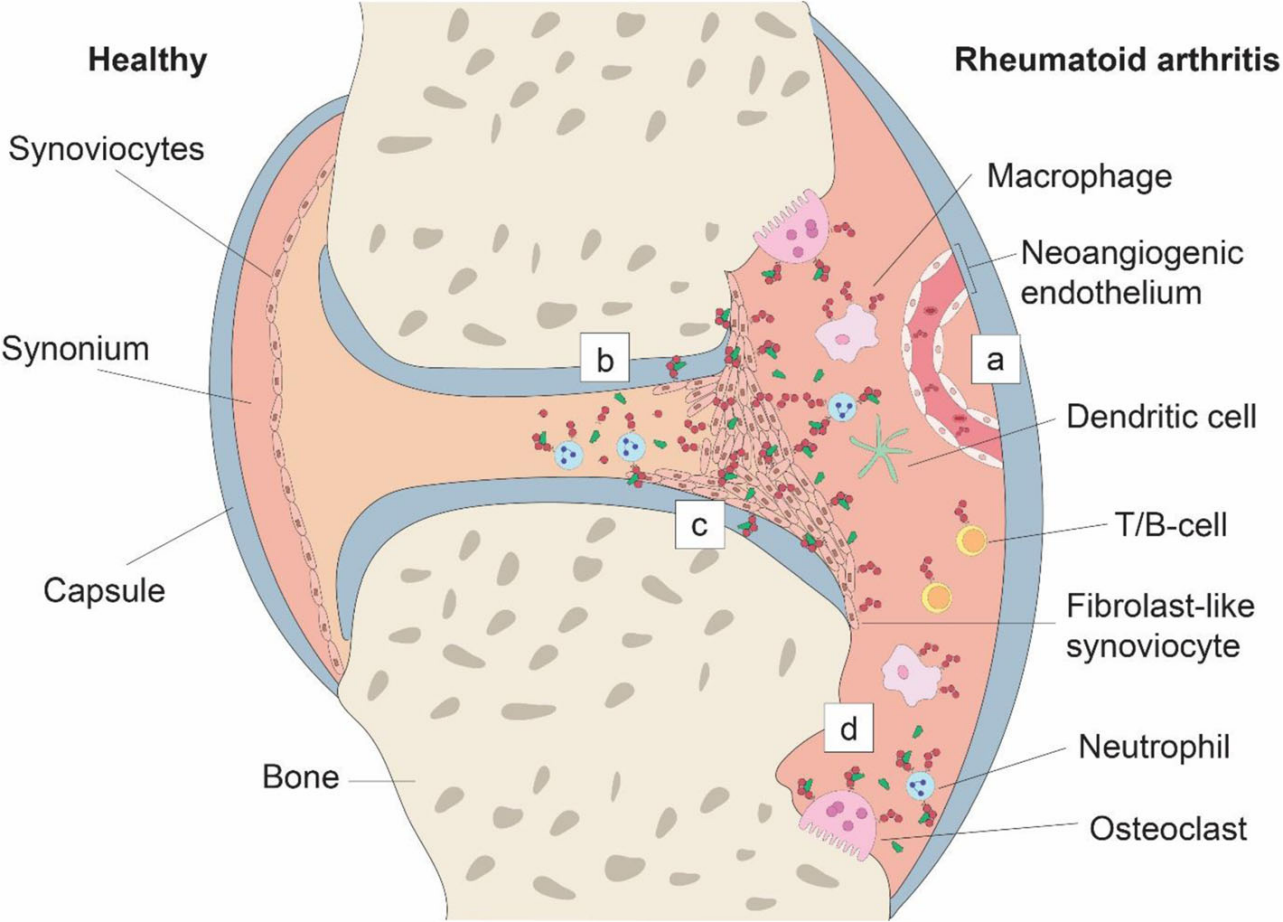
uPAR in rheumatoid arthritis. **a** uPAR on neo-angiogenic endothelium support the influx of inflammatory cells. **b** Neutrophils secrete uPA and uPAR-D2/3 further escalating the inflammation. **c** The uPA interacts via autocrine and paracrine methods with neutrophils, fibroblast-like synoviocytes, macrophages and chondrocytes, activating invasive, and proliferative pathways in these cells. **d** uPAR on osteoclasts promotes bone destruction. uPAR is represented by the red 3-domain structure as described in Fig. 1 on the cell membrane of uPAR expressing cells and uPA by the green structure in the extracellular matrix and bound to uPAR

RA manifestation in joints is defined by persistent synovial inflammation, where leukocytes from the innate and adaptive immune system infiltrate the synovial compartment and interact with present synoviocytes [116]. To support the influx, adhesion, and migration of cells into the synovial compartment, endothelial cells overexpress uPAR (Fig. 3a) [45, 117, 118]. However, uPAR expression is limited to endothelial cells. Neutrophils stimulate the inflammatory process through secretion of uPA and domain 2-3 of uPAR, whereby the latter probably functions as a chemoattractant for other formyl peptide receptor expressing leukocytes (Fig. 3b) [114, 118, 119]. The secreted uPA interacts in autocrine and paracrine fashion with uPAR on neutrophils, FLS, macrophages, and chondrocytes, enhancing the invasive and proliferative properties of these cells (Fig. 3c, d) [16, 115, 120–131]. The importance of uPAR has been confirmed by studies where knockdown of uPAR in FLS-inhibited proliferation, migration, and invasion in vitro [16]. Furthermore, compared to their wildtype littermates, PLAUR−/− mice show significant reduction of arthritis incidence and severity in a collagen-induced arthritis model [132]. However, an earlier study suggested that uPAR is not essential for RA development [133]. Induction of arthritis by intra-articular uPA injection is not dependent on the uPAR-binding fragment of uPA. Furthermore, the arthritis incidence is similar in PLAUR−/− mice and their genetic counterparts after uPA injection [133]. While this model results in joints with morphological features of arthritis, the question can be beckoned if intra-articular injection of uPA accurately reflects the etiology and progression of RA in humans.

Besides influencing the inflammatory stage of RA, uPAR also attenuates the bone destruction occurring in late stage RA disease. Osteoclast differentiation, the subsequent bone destruction and bone mineral density (BMD), is significantly decreased in uPAR knockout mice and stimulated by uPAR overexpression [134]. With this knowledge in mind, loss of BMD has been successfully inhibited in a lipopolysaccharide-induced bone destruction mouse model using the uPAR targeting peptide (Ȧ6) [135].

The therapeutic possibilities by targeting uPAR in vivo have been investigated using uPAR antisense treatment and adenovirus-mediated gene transfer of the amino terminal fragment of uPA fused to human serum albumin. Both inhibit cartilage invasion while the latter also decreases both the incidence and severity of the disease [115, 136, 137]. However, blocking uPAR using the anti-uPAR monoclonal antibody mR1 in collagen-induced and delayed-type hypersensitivity arthritis models has no effect on RA progression [118]. This discrepancy can partly be explained by difference in methods (antisense vs. adenovirus vs. monoclonal antibody administration), targeting uPA vs. uPAR, and by the differences in models used.

While preclinical in vivo research is still inconclusive, several studies with clinically used agents have demonstrated that various treatment options for RA reach their effect by targeting the urokinase plasminogen activation pathway. Tenoxicam, a non-steroidal anti-inflammatory drug, has been shown to downregulate monocyte uPAR expression and hyaluronic acid treatment decreases the immunostaining for uPAR expression on FLS [122, 123]. Furthermore, the widely used corticosteroid deflazacort also modulates the urokinase pathway by inducing PAI-1 and inhibiting uPA and uPAR expression in RA FLS but not in healthy cells [138]. Physiologically, proliferation and invasion of RA FLS are inhibited by deflazacort. In addition, soluble uPAR levels correlate with response to biologicals such as the tumor necrosis factor (TNF)-inhibitor adalimumab [139].

All-in-all there is substantial evidence for the role of uPA/uPAR/PAI-1 axis in RA development and progression. Although future studies will need to confirm this, targeting uPAR for imaging purposes has the potential of providing relevant information on disease activity, prognosis, and treatment effect [140].

### Central nervous system pathology: unraveling pathophysiology of degenerative disease

The nervous system, with the brain as its helm, is the most complex and pivotal system of the human body. Therefore, neurodegenerative disorders, such as Alzheimer’s disease (AD) and Creutzfeld-Jakob disease (CJD), auto-immune diseases, such as multiple sclerosis (MS), and infectious diseases, such as cerebral malaria (CM) and acquired immunodeficiency syndrome dementia complex (ADC), have disastrous consequences for patients. The emergence of molecular imaging has enabled more in-depth research into these pathologies as well as possibilities for diagnosis and monitoring of disease before clinical features occur [141–144].

**Fig. 4:**
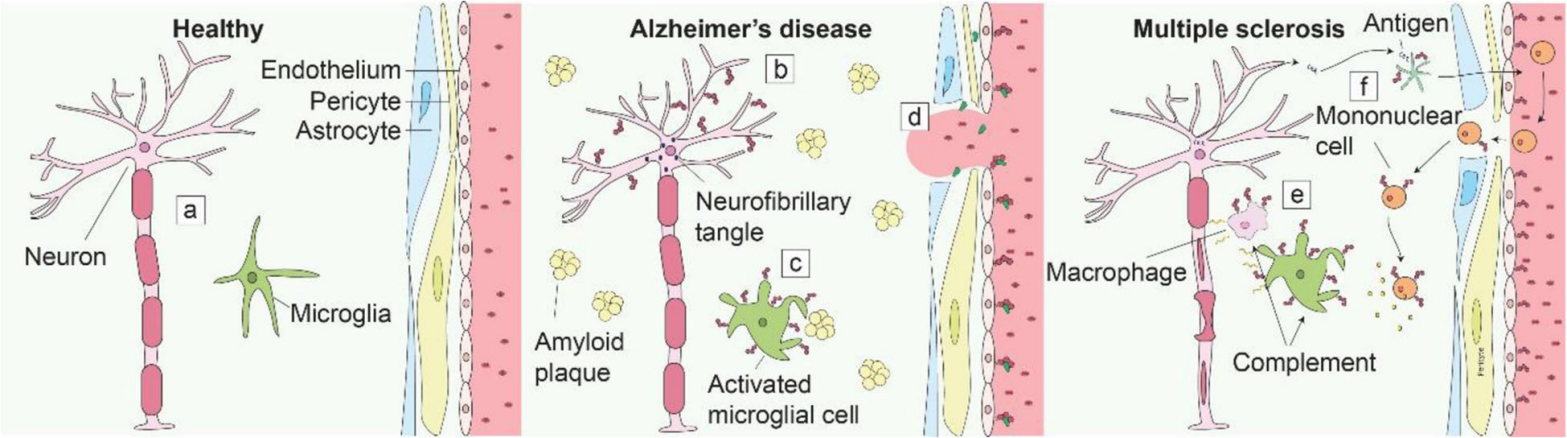
uPAR in degenerative nervous system disorders. **a** uPAR expression is practically absent in the healthy human brain. In Alzheimer’s disease uPAR expression is found on (**b**) cortical neurons, (**c**) activated microglial cells after exposure to the amyloid plaques, and (**d**) in the vascular wall promoting uPA activation, subsequent extracellular matrix breakdown and corresponding spontaneous hemorrhages. In multiple sclerosis, uPAR (**e**) is expressed on inflammatory cells and activated microglial cells promoting local damage. **f** Furthermore, uPAR expression on dendritic cells influences subsequent T cell differentiation. uPAR is represented by the red 3-domain structure as described in Fig. 1 on the cell membrane of uPAR expressing cells and uPA by the green structure in the extracellular matrix and bound to uPAR

While uPAR expression is very low, if not absent, in the adult brain, it plays a pivotal role in the developing brain (Fig. 4a) [15, 145]. In the early brain binding of uPA to uPAR stimulated neuritogenesis, neuronal migration, and differentiation via both proteolytic and nonproteolytic pathways resulting in axonal growth and branching of both the central and peripheral nerves [146–150]. The uPA/uPAR axis is of such importance that dysregulation has been implicated with epilepsy, schizophrenia, and autism. *PLAUR*, the gene encoding uPAR, and its promotor have been found to be upregulated in autistic patients [151, 152]. Furthermore, in rats, uPAR expression was increased in interneurons after spontaneous seizures [153]. On the other hand, uPAR−/− mice were more susceptible to seizures, increased anxiety, and altered social behavior; all characteristics of epilepsy, schizophrenia and autism [154, 155]. The discovery that uPAR functions as a receptor for SRPX2, an important regulator of synapse formation, and that both are co-located both spatially and temporally in the developing brain, further implicates uPAR’s role in the (patho)physiology of the nervous system. Although the actual function of SRPX2 remains to be elucidated, the Y72S mutation in SRPX2 leads to an almost sixfold increased affinity for uPAR, and clinically manifests in seizures, speech deficit, and mental retardation [156, 157].

Various neurodegenerative diseases present with enhanced uPAR expression. AD is the leading cause of dementia and can be characterized pathologically as intracellular tangles and extracellular deposition of amyloid β creating senile plaques [158, 159]. uPAR expression has been found in both the cortical neurons and the vascular wall of AD patients (Fig. 4b) [19, 160–162]. Interestingly, the cerebellum, a region of the brain that is usually not affected by AD, is negative for uPAR in these patients [162]. Corresponding in vitro studies demonstrates that microglia upregulate uPAR mRNA and protein after exposure to aggregated amyloid β (Fig. 4c) [161, 162]. Furthermore, uPA and plasminogen activity is increased, which could lead to the vulnerability of the cerebral vessel wall due to extracellular matrix breakdown and corresponding spontaneous hemorrhages observed in AD (Fig. 4d) [161]. In CJD, another fatal degenerative disease with a mean survival of 7.3+/−0.2 months after clinical onset, significantly more neurons, primarily focused in cortical layer 3-5, express uPAR, where the expression has been associated with damaged neurons as seen by chromatin condensation, hypertrophic swelling, and degeneration [160, 163]. Microglial cells, but not astrocytes, also express uPAR [160].

MS is an autoimmune disease where an immune response is mounted against the central nervous system by autoreactive lymphocytes resulting in lesions that are characterized by inflammation, demyelination, and degeneration of neurons [164]. While autopsy material from healthy brains exhibits almost no uPAR expression, uPAR gene and protein expression are significantly elevated on MS microvessels, mononuclear cells, macrophages, pericytes, and smooth muscle cells [165–169]. Microglial cells cultured from an MS patient show an activated morphology in combination with high levels of uPAR, whereas control microglial cells from normal brain tissue express little to no uPAR mRNA and protein. After in vitro activation, these normal microglia present a spindle-shape morphology and express uPAR [170]. In an animal model of experimental autoimmune encephalomyelitis (EAE), elevated uPAR expression is detected in the inflammatory lesions by both immune and microglial cells (Fig. 4e) and increased uPA activity at the dorsal horn and central spinal cord [171, 172]. EAE in uPAR−/− mice is characterized by a delayed onset, chronicity, persisting inflammatory cuffs with increased levels of uPA and more extensive demyelination. The dysregulated adhesion and migration of inflammatory cells in uPAR−/− mice explains the delayed onset while the inability to recycle uPA via uPAR reflects the increased neuronal damage [173]. In a later study, uPAR−/− mice with EAE are shown to exhibit more severe disease with a twofold increase in microglial activation and increased infiltration of mononuclear cells but reduced immune response, rendering the mouse incapable of recovery [174]. The recently revealed crosstalk between the coagulation pathway (coagulation factor XII, FXII) and immunity in MS underlines the role of uPAR in this disease. uPAR on dendritic cells (Fig. 4f) is responsible for the immune modulatory function of FXII, tipping the balance of T cell differentiation toward the TH17 phenotype, as a signal receiver and relaying the message, via CD11b integrin, intracellularly (Fig. 4f) [175]. All-in-all, there is initial evidence that uPAR plays a fundamental role in MS, but whether uPAR expression is protective or destructive remains to be elucidated and, considering uPAR’s multifaceted aspects, could actually be both.

Various infectious diseases can have drastic neurological manifestations. ADC is one of the most severe consequences of human immunodeficiency virus 1 (HIV-1) infection [176]. The lesions showed membranous uPAR expression in immunohistochemical stainings that colocalized with HIV-1 p24 antigen in both macrophages, microglial, and multinucleated giant cells [177, 178]. Not coincidentally, soluble uPAR levels are a strong independent predictor for HIV-1 infection survival [179]. While combination antiretroviral therapy has successfully dropped the incidence of ADC from 20 to 5%, milder forms of HIV-associated neurocognitive disorder still occur with an incidence of 20-50% [176]. No study has evaluated uPAR in these cases. Plasmodium falciparum is another infectious agent that can lead to severe neurologic impairment with persistent neurocognitive deficits characterized as CM [180]. In post-mortem specimens of patients with CM uPAR expression, detected by immunohistochemical staining, of microglia, reactive astrocytes and endothelial cells is limited to areas with microvasculature containing parasitized erythrocytes, petechial bleedings and Dürck’s granulomas [181]. In the mouse model of CM, known as severe malaria (SM) as the syndrome in mice is not limited to the brain, uPAR deficiency has profound effect on thrombocytopenia. Platelet trapping, which is a reliable predictor of forthcoming death, does not occur in uPAR−/− mice [182]. The current theory holds that platelets form an adhesive surface in microvascular beds for parasitized erythrocytes in the cerebrum and consequently play a pivotal role in the development of CM [183].

While in most neurological disease processes, there is no clear indication whether uPAR expression is protective or destructive, the evidence currently accumulated suggests a critical role for uPAR in the pathophysiology of AD, MS, ADC, and MC. Grossly, aberrant uPAR expression is linked to an altered immune-phenotype, consequently altering the progressing of the disease. In addition to the post-mortem pathology and animal models, we are dependent on for research, an uPAR targeting tracer may enable in vivo imaging of the various pathophysiological processes going on in real-time and consequently enrich our understanding of these disease. This knowledge can potentially be used to dictate treatment and monitor disease based on uPAR signaling.

### Inflammatory bowel disease: imaging macrophage polarization

Inflammatory bowel disease (IBD) is an umbrella term consisting of chronic relapsing inflammatory disorders of the intestinal tract. Ulcerative colitis (UC) characterizes itself as inflammation of the mucosal layer of the colon while Crohn’s disease (CD) displays transmural inflammation of any part of the gastrointestinal tract ranging from the mouth to the anus [184]. The current gold standard for diagnosis and surveillance of IBD is endoscopy and X-ray exams, but these techniques are limited by their invasiveness and patient tolerance. Molecular imaging might provide an opportunity for accurate non-invasive or endoscopic specification of IBD presence, transmural and extra-intestinal tissue involvement, and specific inflammatory profile [185–188]. While the etiology of IBD has not been fully elucidated yet, genetic, environmental, and immune factors have all been implicated.

**Fig. 5:**
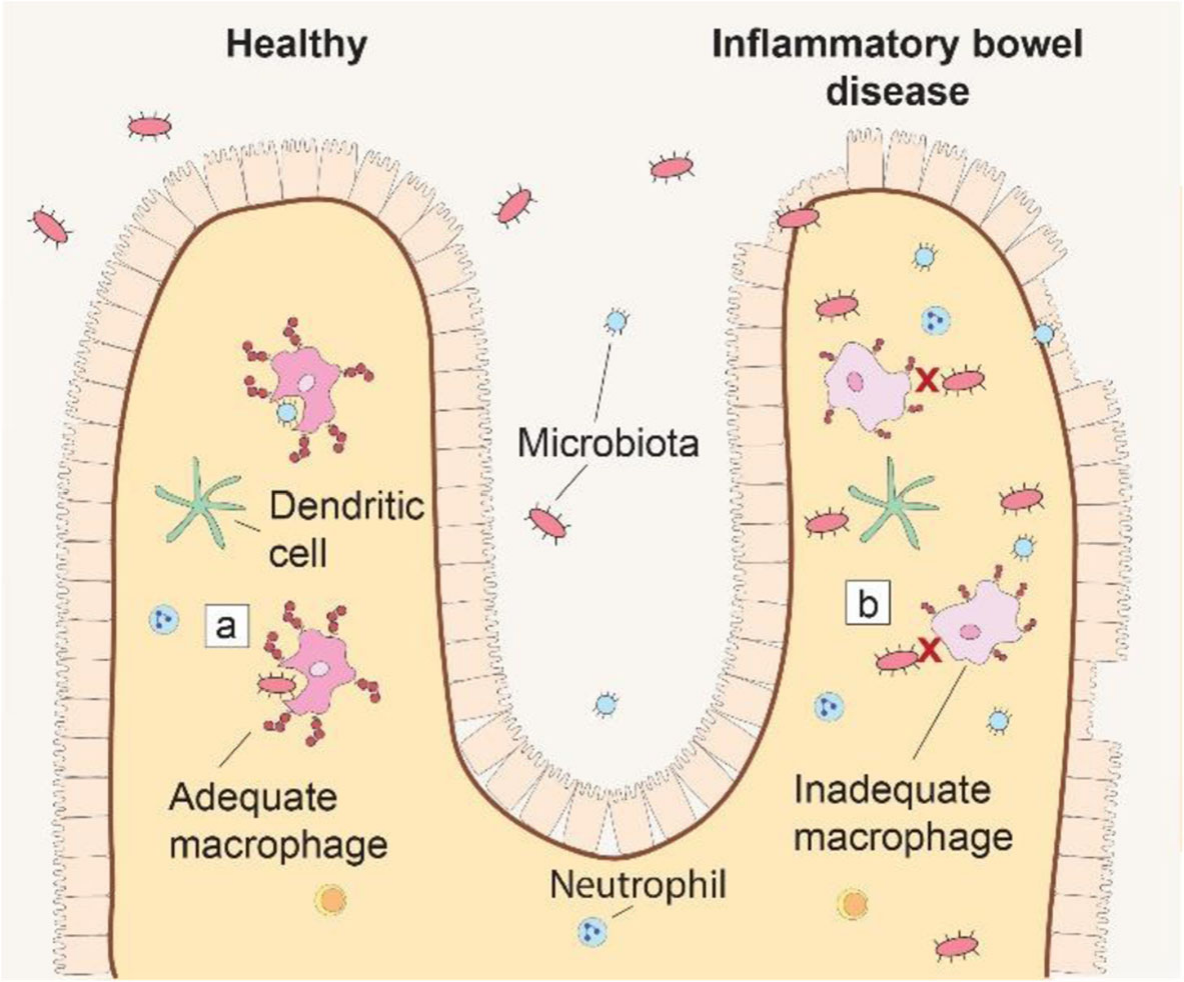
uPAR in inflammatory bowel disease. **a** Macrophage uPAR D1-D3 expression plays a significant role in the bacterial removal while (**b**) in inflammatory bowel disease macrophage differentiation is altered with as consequence an increase in uPAR D2-D3 expression and inadequate microbial maintenance. uPAR is represented by the red 3-domain structure as described in Fig. 1 on the cell membrane of uPAR expressing cells

The impaired immune response leads to extensive tissue remodeling and degradation in which the plasminogen activation cascade, including various MMPs and localized by uPAR, plays a major role [189–192]. Patients with active IBD have increased uPAR specific for macrophages at active lesions. Interestingly, uPAR D1-D3 is downregulated while uPAR D2-D3 is increased. In two different IBD mice models, uPAR expression has shown specific for CX_3_CR_1_^+^ macrophages and mirrored disease onset [193]. This subset of macrophages has an anti-inflammatory phenotype [194]. Therefore, knocking out uPAR exaggerates disease by amplifying the release of pro-inflammatory cytokines and altering polarization of macrophages. Low expression of uPAR D1-D3 and high expression of uPAR D2-3 by IBD patients can consequently lead to increased inflammation and disrupted bacterial removal (Fig. 5a, b) [193]. The therapeutic potential that targeting macrophages, and in extension uPAR, brings has not been unnoticed [195]. A cyclic peptide based of amino acids 88-92 of uPAR, [SRSRY], competed with uPAR for binding to FPRL1 but exerted an opposite effect: inhibiting migration as opposed to promoting it [196]. In vivo, [SRSRY], altered macrophage polarization and migration in colitis mice models and as such attenuated disease severity [197]. By competing with the migration sensitive epitope that becomes available after uPAR cleavage, [SRSRY], diminishes the destructive potential of uPAR D2-D3. While the research is still in its infancy, there is potential to determine macrophage polarization and disease progression by molecular imaging of uPAR. Determining the right epitope to direct the uPAR targeting moiety to, will be crucial for correct implementation and interpretation of uPAR-targeted molecular imaging in IBD as well as for other applications (Table 1). If addressed well, uPAR imaging has the potential to non-invasively diagnose IBD by identifying aberrant macrophage polarization and subsequently be used to monitor disease activity.

**Table 1:** uPAR targeting imaging agents

Agent	Classification	Targeting epitope	Imaging modality	Imaging window	Notes	Translation stage	Ref
Cy5.5-mATF-IO	ATF-based NP	uPA-binding region	MRI, optical	24-48 h	Mouse ATF	In vivo preclinical	[198, 199]
hATF-Cy5.5-IO-Nos	ATF-based NP	uPA-binding region	MRI, optical	n.v.t.	Human ATF	In vitro preclinical	[200]
ATF-I^125^	ATF-based	uPA-binding region	n.a.	n.v.t.		In vitro preclinical	[201]
NIR-830-mATF-IONP	ATF-based NP	uPA-binding region	PA, optical	24 h	Mouse ATF	In vivo preclinical	[202]
ATF-IONP-Gem	ATF-based NP	uPA-binding region	MRI	48 h	Mouse and human ATF	In vivo preclinical	[203]
NIR-830-hATF-IONP	ATF-based NP	uPA-binding region	Optical	24 h	Human ATF	In vivo preclinical	[204]
NAc-dD-CHA-F-dS-dR-Y-L-W-S-βAla)_2_-K-K(DOTA)-NH_2_-^111^In	Peptide	uPA-binding region	n.a.	n.v.t.		In vitro preclinical	[201]
^99m^Tc-Hynic-PEG-AE105	Peptide	uPA-binding region	SPECT	4-6 h		In vivo preclinical	[205]
^64^Cu-DOTA-AE105	Peptide	uPA-binding region	PET	24 h		Phase I clinical	[13, 219–222, 224]
^68^Ga-NOTA-AE105	Peptide	uPA-binding region	PET	10 min-1 h		Phase I clinical	[14, 206]
ICG-Glu-Glu-AE105	Peptide	uPA-binding region	Optical	6-24 h		In vivo preclinical	[207–209]
CH1055-4Glu-AE105	Peptide	uPA-binding region	Optical	72-96 h		In vivo preclinical	[210]
AF680-2G10	Antibody	uPA-binding region	Optical	48-96 h	Recombinant antibody with trastuzumab Fc region	In vivo preclinical	[211, 212]
^111^ln-2G10	Antibody	uPA-binding region	SPECT	48-120 h	Recombinant antibody with trastuzumab Fc region	In vivo preclinical	[211, 212]
AF680-3C6	Antibody	β1-binding region	Optical	48-96 h	Recombinant antibody with trastuzumab Fc region	In vivo preclinical	[211]
^111^ln-3C6	Antibody	β1-binding region	SPECT	48-96 h	Recombinant antibody with trastuzumab Fc region	In vivo preclinical	[211]
^111^In-ZW800-1-ATN-658 (Hybrid ATN-658)	Antibody	Domain 3, amino acids 268-275	Optical, SPECT	24-72 h	Mouse antibody	In vivo preclinical	[213, 214]

*NP* nanoparticle, *MRI* magnetic resonance imaging, *PET* positron emission tomography, *SPECT* single photon-emission computed tomography, *h* hours, *min* minute, *n.a.* not applicable, *ATF* amino-terminal fragment

### uPAR imaging

**Fig. 6:**
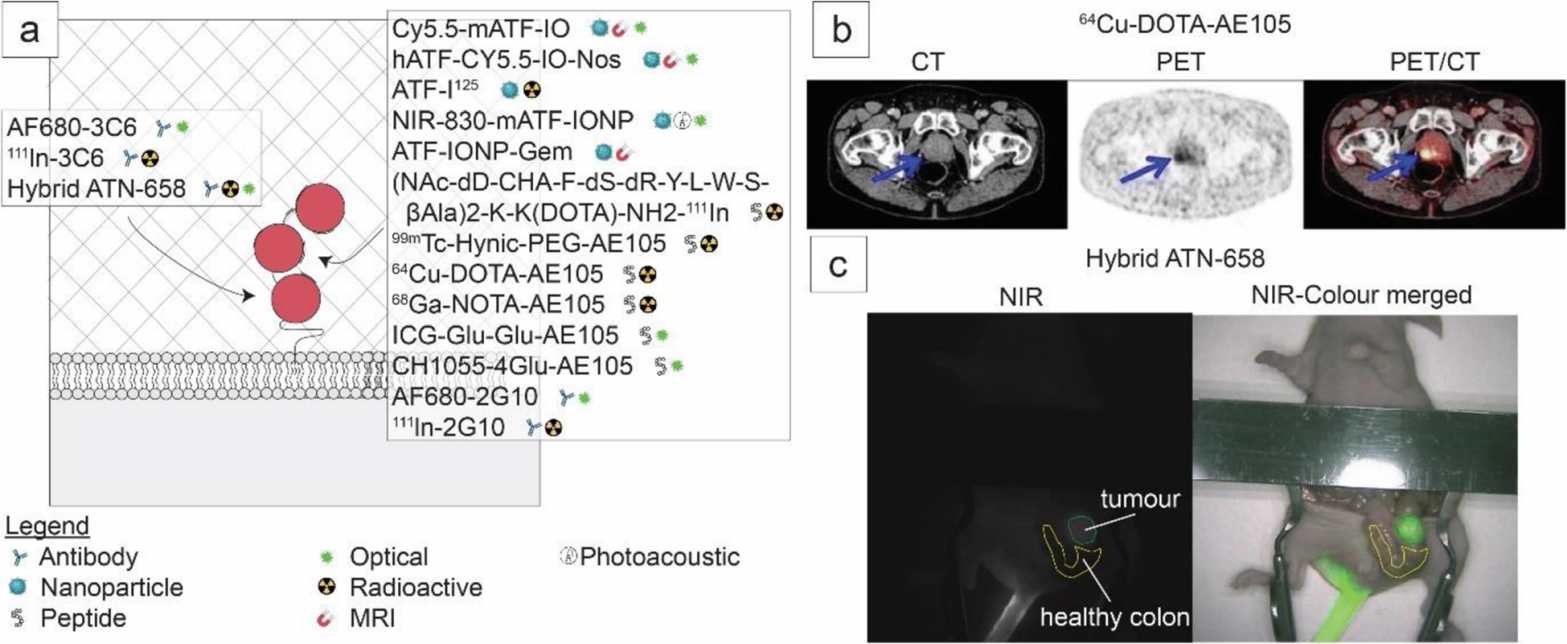
Targeting uPAR for molecular imaging. **a** Representation of binding domains of the uPAR imaging agents currently under development, their classification and suitable imaging modality. **b** Positive primary lesion with uPAR PET in human prostate cancer after injection of 64Cu-DOTA-AE105. Images adapted from Skovgaard et al. [218] and used under the terms of the Creative Commons CC BY license. **c** NIR optical imaging of orthotopic colon cancer with hybrid ATN-658

uPAR has been targeted for molecular imaging according by various approaches, each with its own advantages and disadvantages (Table 1, Fig. 6a). The first peptides targeting uPAR were ligand-based, utilizing the growth-factor domain of urokinase [215]. Targeting this natural interaction between uPA and uPAR with ATF or ATF-like constructs has been employed for magnetic-resonance imaging, near-infrared imaging, photo-acoustic imaging, and nuclear-imaging [198–204]. With a molecular weight of 18.5 kilodalton, ATF is cleared rapidly by the kidneys resulting in quick imaging times (30 min to 2 h) but also minimizing the time available to get sufficient contrast [216]. Conjugating ATF to nanoparticles (NPs) enhances blood circulation times resulting in optimal imaging times around 24-48 h after injection in vivo [198, 199, 202, 204]. Another advantage of ATF-NPs is their internalization, potentially increasing contrast [199, 200, 204]. Nonetheless, whether conjugated to a NP or not, uPAR targeting efficiency with ATF is dependent on the absence of endogenous urokinase on the majority of uPAR copies present and markedly reduced in models with high uPA expression [5, 201].

An alternative uses a 9-mer peptide which has led to the first uPAR PET clinical trials. This peptide, AE105, is the refined version of a 15-mer peptide identified by a phage display with uPAR-transfected cell lines and binds uPAR at the uPA-binding site in a species specific manner, like ATF [217, 218]. While AE105 has also been conjugated with (radio)-labels for single-photon emission computed tomography (SPECT) and near-infrared fluorescence (NIRF) in preclinical oncology studies, this section will focus on positron-emission tomography as AE105 PET is further along the clinical pipeline [205–210, 219, 220]. Initially, AE105 has been conjugated with the metal chelator DOTA and subsequently labeled with ^64^Cu. ^64^Cu-DOTA-AE105 specifically targets uPAR positive lesions in preclinical studies with signal corresponding to uPAR expression levels and epitope availability, but also resulted in high non-specific liver-uptake [221, 222]. Alternative ^64^Cu, ^68^Ga, and ^18^F tracer-chelator combinations decrease non-specific uptake but at the cost of lower tumor specific signal [223]. Phase I clinical trials with ^64^Cu-DOTA-AE105 have shown no adverse events or detectable pharmacological effects related to the tracer. Furthermore, all primary tumors (bladder, breast, and prostate) and the majority of metastasis are identifiable between 1 and 24 h after administration (Fig. 6b). In this study, two liver metastasis have not been visualized due to high background signal [13]. In addition, the feasibility of measuring mean ^64^Cu-DOTA-AE105 uptake in the arterial beds of these patients in order to non-invasively identify atherogenic lesions has been retrospectively evaluated [224]. While activated macrophages have higher uPAR expression, clear imaging capability of atherosclerosis has yet to be demonstrated with this tracer. The possibility to scan at early time points and the independence of ^68^Ga on an on-site cyclotron prompted to phase I trials of ^68^Ga-NOTA-AE105 [13, 14, 223]. ^68^Ga-Nota-AE105 resulted in decreased liver signal and specifically identified both primary tumors and one metastasis missed in the standard work up [14]. While initial clinical trial results are promising, allowing for rapid identification of cancerous lesions, endogenous uPA expression could present the biggest limiting factor of AE105 molecular imaging, especially in diseases where the expression of uPA is likely to be increased and paramount for outcomes [5, 225].

Another approach utilizes monoclonal antibodies to target uPAR. Both antibodies 2G10 and 3C6 are identified from a human fragment of the antigen binding (Fab) phage display library to have high affinities for uPAR. Consequently, these are expressed as recombinant IgG’s using the trastuzumab Fc domain [226]. 2G10 competes with uPA for uPAR binding while 3C6 prevents β1 integrin association with uPAR [211]. In human xenograft breast cancer models, 2G10 shows higher tumor uptake with NIRF and SPECT/CT than 3C6, probably due to higher epitope availability for 2G10 [212, 226]. Another thoroughly and extensively studied anti-uPAR antibody is ATN658. ATN658 was raised against a soluble D2-D3 uPAR fragment and recognizes domain 3 of uPAR, close to the C-terminus at amino acids 268-275 [47, 227]. ATN658 enables and anti-tumor effect by impairing α5β1 integrin adhesion to the ECM and is not effected by uPA or vitronectin interaction with uPAR [47, 228, 229]. In colorectal and oral xenograft cancer models NIRF and SPECT hybrid-labeled ATN658, accurately localized lesions as small as 1-2 mm in size in a range from 24 to 72 h post-injection (Fig. 6c) [213, 214]. ATN658 has been humanized and is awaiting clinical translation for NIRF-imaging [229].

A thorough assessment of the uPAR targeting agents reveals crucial differences in modalities, biodistributions, imaging windows, epitopes targeted, and production methods. Therefore, a one-size-fits-all solution to target all types of diseases where uPAR is involved is probably not feasible, like for most, if not all, molecular targets [5]. For instance, peptides may find their utility in more acute situations such as atherosclerosis imaging. Antibodies seem more ideal for abdominal imaging where the high non-specific background of kidneys can be a hindrance or in more elective settings where a large imaging window is desired. Not only will selecting an optimal agent be challenging, also designing and selecting preclinical animal models that take the species specificity of the imaging agents into account, since most tracers designed for clinical applications have high affinities for human uPAR but no or reduced affinities for mouse uPAR [201, 218, 226, 227].

## Conclusions

uPAR is a central unit in regulating ECM proteolysis, migration, differentiation, and proliferation and hereby implicated in a range of inflammatory-related diseases, often holding pivotal roles and tipping the balance toward disease aggravation. Even though uPAR is almost completely absent in normal tissue, it will likely not be an appropriate target for the diagnosis of diseases, due to the common pathophysiological role. However, when it comes to visualization of diagnosed disease lesions, whether it be plaques that are about to rupture or aggravation of RA or IBD, uPAR plays a central pathophysiological role prompting its usefulness as a molecular imaging target. Furthermore, molecular imaging of uPAR can unravel the complex pathophysiological processes occurring, increasing our understanding of the disease, and consequently allowing the development of novel therapies, ultimately improving patient outcomes.

## Data Availability

Not applicable.

## Abbreviations

AD: Alzheimer’s disease
ADC: Acquired immunodeficiency syndrome dementia complex
ATF: Amino-terminal fragment of urokinase
BMD: Bone mineral density
BPTI: Bovine pancreas trypsin inhibitor
CD: Crohn’s disease
CJD: Creutzfeld-Jakob disease
CM: Cerebral malaria
D: Domain: D1, D2, D3, D1-3, D1-2, D2-3
ECM: Extracellular matrix
FLS: Fibroblast-like synoviocytes
FPRL1: Formyl peptide receptor-like 1
GPI: Glycosylphosphatidylinositol
GPCR: G protein-coupled receptors
HIV-1: Human immunodeficiency virus 1
IBD: Inflammatory bowel disease
LRP1: Lipoprotein receptor-related protein 1
MMP: Matrix metalloprotease
MS: Multiple sclerosis
NIRF: Near-infrared fluorescence
NP: Nanoparticle
PAI-1: Plasminogen activator inhibitor type 1
PET: Positron emission tomography
RA: Rheumatoid arthritis
SM: Severe malaria
SPECT: Single-photon emission computed tomography
TIMP1: Tissue inhibitor of matrix metalloproteinases 1
TNF: Tumor necrosis factor
UC: Ulcerative colitis
uPA: Urokinase plasminogen activator
uPAR: Urokinase plasminogen activator receptor
VSMC: Vascular smooth muscle cell
